# Mid-term functional outcomes and graft survivorship after allograft ACL reconstruction in recreationally active adults aged 40–60 years

**DOI:** 10.1016/j.asmart.2026.10.002

**Published:** 2026-11-07

**Authors:** Horacio Rivarola, Francisco Endara Urresta, Cristian Collazo, Marcos Palanconi, Marcos Meninato, Carlos Peñaherrera-Carrillo, Alejandro Barros Castro, Brian Flores Agreda, Bautista Rivarola

**Affiliations:** a Hospital Universitario Austral, Buenos Aires, Argentina; b Clínica Arthros, Quito, Ecuador; c Instituto Nacional de Rehabilitación, Mexico City, Mexico; d Universidad Internacional del Ecuador, Quito, Ecuador

**Keywords:** Anterior cruciate ligament, Allograft, Graft survivorship, Non-irradiated frozen allograft, Recreational athletes, Return to sport

## Abstract

**Background:**

An increasing proportion of anterior cruciate ligament (ACL) reconstructions are performed in older recreationally active adults; however, mid-term functional and graft survivorship data remain limited for this specific population.

**Purpose:**

To evaluate mid-term functional outcomes, return to recreational sport, and graft survivorship following primary allograft ACL reconstruction in recreationally active adults aged 40–60 years.

**Methods:**

A retrospective cohort study included 300 recreationally active adults aged 40–60 years who underwent primary allograft ACL reconstruction between 2015 and 2019. All grafts were non-irradiated and preserved by freezing according to the tissue bank protocol. Validated patient-reported outcome measures (IKDC, KOOS, Tegner) were collected preoperatively and at final follow-up. Return to sport was defined as sustained participation in pre-injury recreational activity. Graft failure was defined by recurrent instability, imaging-confirmed rupture, or revision surgery. Kaplan–Meier analysis was used to assess graft survivorship.

**Results:**

At a mean follow-up of 62.4 ± 9.1 months, significant improvements were observed across all functional outcome measures. The mean IKDC score improved from 46.2 ± 11.8 preoperatively to 82.4 ± 10.6 at final follow-up, and the mean KOOS overall score increased from 58.7 ± 13.4 to 86.1 ± 9.8. Documented resumption of recreational sport participation occurred in 87.3% of patients at a mean of 8.9 ± 2.4 months postoperatively. Kaplan–Meier graft survivorship was 98.3% at 2 years, 96.7% at 3 years, and 94.8% at 5 years. Sixteen graft failures (5.3%) were recorded.

**Conclusion:**

In recreationally active adults aged 40–60 years, primary allograft ACL reconstruction was associated with improvement in patient-reported outcomes, frequent resumption of recreational sport participation, and satisfactory graft survivorship at mid-term follow-up.

**Level of evidence:**

Level III, retrospective cohort study.

**What's New**

• This study provides mid-term functional and graft survivorship data in recreationally active adults aged 40–60 years undergoing primary allograft anterior cruciate ligament reconstruction.
• Functional recovery, return to recreational sport, and graft survivorship are reported using standardized definitions and validated outcome measures.
• The findings characterize outcomes within a standardized allograft-processing cohort without making comparative claims regarding graft source or processing method.

## 1 Introduction

The clinical profile of patients undergoing anterior cruciate ligament (ACL) reconstruction has progressively shifted over recent years. Beyond the traditional cohort of young competitive athletes, an increasing number of procedures are now performed in middle-aged individuals who remain physically active and seek to maintain participation in recreational sports. Older recreationally active adults undergoing anterior cruciate ligament reconstruction represent a clinically distinct population from the younger competitive cohorts that dominate much of the available literature. For the present study, an age interval of 40–60 years was prespecified to examine outcomes in recreationally active adults beyond the age range most commonly represented in young-athlete anterior cruciate ligament reconstruction studies, while limiting the potential influence of more advanced age-related degenerative joint disease. For this population, expectations after surgery are distinct from those of elite athletes: the primary goals are restoration of knee stability, predictable functional recovery, and sustained ability to engage in recreational physical activity over time rather than maximal performance. As life expectancy and activity levels continue to rise, addressing the specific needs of this patient group has become increasingly relevant in contemporary sports medicine practice. 1, 2, 3, 4.

Graft selection in ACL reconstruction remains a topic of ongoing discussion, particularly in middle-aged patients. Autograft tissue is widely regarded as a reliable option, especially in younger and high-demand athletes; however, donor-site morbidity, postoperative anterior knee pain, and prolonged recovery may represent meaningful drawbacks in older recreational athletes. Allograft tissue offers potential advantages in this context, including reduced surgical morbidity, shorter operative time, and avoidance of harvest-related complications. At the same time, concerns persist regarding biological incorporation, delayed remodeling, and graft failure, particularly when grafts are subjected to irradiation or aggressive processing. Allograft processing is an important methodological consideration because irradiation and chemical sterilization may alter the biological and mechanical properties of tendon tissue and have been associated with differences in clinical outcomes 3, 4, 5, 6. However, non-irradiated frozen or fresh-frozen grafts are routinely used in many contemporary tissue-banking and sports medicine settings. Accordingly, the present study did not investigate non-irradiated freezing as a novel processing strategy or compare alternative preservation methods. Rather, graft processing was standardized across the cohort to reduce processing-related variability and to characterize outcomes within a consistently treated population. 3, 4, 5, 6.

Despite the growing use of non-irradiated frozen allografts in selected patients, mid-term outcome data remain limited for recreationally active adults beyond the younger high-demand populations that predominate in anterior cruciate ligament reconstruction research. The 40–60-year age interval was therefore selected a priori as an operational study range rather than as a universal definition of middle age, with the aim of evaluating a clinically relevant group of older recreational athletes while applying consistent age boundaries across the cohort.

Most available studies either combine heterogeneous age groups or focus on short-term results, making it difficult to draw meaningful conclusions for this specific population. The purpose of the present study was therefore to evaluate mid-term functional outcomes, return to recreational sport, and graft survivorship following primary ACL reconstruction using non-irradiated frozen allograft tissue in recreational athletes aged 40–60 years. By focusing on a well-defined cohort and clinically relevant endpoints, this study aims to provide pragmatic data to inform graft selection and patient counseling in routine clinical practice.

## 2 Methods

### 2.1 Study design

This study was designed as a retrospective cohort analysis evaluating mid-term clinical outcomes following primary anterior cruciate ligament (ACL) reconstruction using non-irradiated frozen allograft tissue. The study was conducted at a high-volume sports medicine center specializing in knee ligament surgery. Institutional review board approval was obtained prior to data collection, and all procedures were performed in accordance with ethical standards for clinical research. The level of evidence was classified as Level III. Patients were consecutively included over a predefined enrollment period extending from January 2015 to December 2019, allowing for a minimum clinical follow-up of 5 years for all eligible participants.

### 2.2 Patient selection

Electronic medical records were reviewed to identify patients who met the inclusion criteria. Patients were eligible if they were between 40 and 60 years of age at the time of surgery and had undergone primary ACL reconstruction using non-irradiated frozen allograft tissue. The 40–60-year age interval was prespecified as an operational study criterion and was not intended to represent a universal epidemiological definition of middle age. The lower cutoff was selected to focus the analysis beyond the younger athletic populations in whom graft failure risk and graft-selection considerations have been most extensively studied, whereas the upper cutoff was intended to reduce the potential contribution of increasing age-related degenerative joint disease to functional outcomes. Advanced tibiofemoral osteoarthritis remained an independent exclusion criterion.

Additional eligibility criteria included regular participation in recreational sporting activity before injury and a documented pre-injury Tegner activity score between 4 and 7. All patients had a diagnosis of complete ACL rupture confirmed by clinical examination and magnetic resonance imaging and reported symptomatic instability that limited daily activities or recreational sports participation despite structured nonoperative management.

For the purposes of this study, recreational athletes were defined as individuals who regularly participated in organized or non-organized sports before injury but were not professional, collegiate, or elite competitive athletes. To provide an objective activity-based criterion and reduce heterogeneity in functional demand, eligibility was additionally restricted to patients with a pre-injury Tegner activity score of 4 to 7. This range was selected to represent recreationally active individuals participating in regular sporting activities while excluding patients with predominantly sedentary or low-demand activity profiles and those competing at the highest activity levels. Pre-injury Tegner activity level was determined from the prospectively documented clinical history and activity assessment available in the medical record.

Exclusion criteria included revision ACL reconstruction, multiligamentous knee injuries, concomitant grade III collateral ligament injuries requiring surgical repair, inflammatory joint disease, advanced tibiofemoral osteoarthritis (Kellgren–Lawrence grade ≥3), prior ipsilateral knee arthroplasty, or incomplete clinical or follow-up data.

### 2.3 Surgical technique

All procedures were performed arthroscopically by fellowship-trained sports medicine surgeons using standardized principles of anatomic ACL reconstruction. Femoral and tibial tunnel placement was individualized based on native ACL footprint anatomy, with the goal of restoring physiological graft orientation and knee stability. Tunnel drilling techniques and fixation strategies were selected according to surgeon preference and intraoperative considerations, including bone quality and graft dimensions. Fixation methods included interference screw fixation and/or cortical suspensory devices, without emphasis on a single fixation construct. Concomitant meniscal tears were treated at the time of ACL reconstruction according to tear pattern and tissue quality, using repair or partial meniscectomy when indicated.

### 2.4 Graft characteristics

All reconstructions were performed using allograft tissue obtained from accredited tissue banks compliant with national regulatory standards. Graft processing was consistent across the cohort: no terminal irradiation or chemical sterilization was applied, and all grafts were preserved in a frozen state according to the corresponding tissue bank protocol. Grafts were thawed intraoperatively according to the manufacturer's or tissue bank's recommended protocol. The study was not designed to compare graft-processing or preservation methods. Allograft tissue type and prepared graft diameter were documented intraoperatively for all cases.

Allograft tissue type and prepared graft diameter were documented intraoperatively for all cases.

### 2.5 Rehabilitation protocol

All patients followed a standardized postoperative rehabilitation program supervised by experienced physical therapists. The protocol emphasized early restoration of knee range of motion, progressive weight-bearing as tolerated, and quadriceps activation during the initial postoperative phase. Subsequent phases focused on neuromuscular control, strength development, and gradual reintroduction of sport-specific activities. Return to running and progressive sport-specific activity were guided clinically by recovery of range of motion, strength, movement control, absence of symptomatic instability, and patient-reported confidence rather than by a fixed postoperative time point. A uniform validated return-to-sport testing battery or psychological readiness instrument was not systematically used across the study period. No accelerated or graft-specific rehabilitation modifications were employed.

### 2.6 Outcome measures

Clinical outcomes were assessed using validated patient-reported outcome measures collected preoperatively and at final follow-up. Functional outcomes included the International Knee Documentation Committee (IKDC) subjective knee form and the Knee injury and Osteoarthritis Outcome Score (KOOS), assessed preoperatively and at final follow-up. Activity level was assessed using the Tegner activity scale, with the pre-injury score used to characterize baseline recreational sporting demand and the final follow-up score used to evaluate postoperative activity recovery. Clinical relevance of the change in IKDC subjective score was additionally assessed using the minimal clinically important difference (MCID). The MCID was not derived from the present cohort; instead, a previously published threshold of [INSERT EXACT VALUE] points for the IKDC subjective score was applied. Individual change scores were calculated by subtracting the preoperative IKDC score from the final follow-up score, and patients with an improvement equal to or greater than the prespecified MCID threshold were classified as achieving a clinically meaningful improvement.

Return to recreational sport participation was operationally defined as documented resumption of recreational sporting activity for at least three consecutive months without interruption attributable to symptomatic instability or functional limitation of the operated knee. This definition was intended to capture sustained sport participation rather than formal return-to-sport clearance or readiness. Objective performance testing, limb-symmetry criteria, and validated psychological readiness measures were not systematically available in the retrospective records and therefore were not incorporated into the return-to-sport outcome definition. Time to RTS and level of activity achieved were recorded. Postoperative complications, including graft failure, reoperations, stiffness, infection, and thromboembolic events, were systematically documented.

### 2.7 Graft failure definition

Graft failure was defined using strict criteria and considered present if one or more of the following occurred: (1) recurrent symptomatic instability confirmed by clinical examination; (2) radiological evidence of graft rupture on magnetic resonance imaging; or (3) need for revision ACL reconstruction. Isolated subjective symptoms without objective findings were not classified as graft failure.

### 2.8 Statistical analysis

Statistical analyses were performed using R software, version 4.4.2 (R Foundation for Statistical Computing, Vienna, Austria). Continuous variables were summarized as mean ± standard deviation and range, and categorical variables were reported as frequencies and percentages. Distributional assumptions for continuous outcome variables were assessed using the Shapiro–Wilk test and visual inspection of quantile–quantile plots. Preoperative and final follow-up IKDC and KOOS scores were compared using paired-samples t tests when the distribution of within-patient differences was approximately normal and Wilcoxon signed-rank tests when normality assumptions were not met. The Tegner activity score was analyzed using the Wilcoxon signed-rank test because of its ordinal structure. All statistical tests were two-sided, and a p value < 0.05 was considered statistically significant.

Individual change in the IKDC subjective score was calculated as the final follow-up score minus the preoperative score. Achievement of the minimal clinically important difference (MCID) was defined as an individual improvement of at least 11.5 points, based on the prespecified published threshold. MCID achievement was reported as the number and percentage of patients meeting or exceeding this threshold.

Graft survivorship was estimated using the Kaplan–Meier method, with graft failure as the event of interest. Patients without graft failure were censored at the date of their last documented clinical assessment. Survivorship estimates at 2, 3, and 5 years are reported with corresponding 95% confidence intervals. To explore whether graft failure was independently associated with baseline or surgical characteristics, a penalized Cox proportional hazards regression model was performed. Age at surgery, sex, pre-injury Tegner activity score, concomitant meniscal procedure, and prepared graft diameter were entered as prespecified covariates. Penalization was used because of the limited number of graft-failure events and to reduce coefficient instability and overfitting. Adjusted associations are reported as hazard ratios with 95% confidence intervals. The proportional hazards assumption was assessed using Schoenfeld residuals.

Patients with incomplete baseline or outcome data required for the primary analyses were excluded according to the prespecified eligibility criteria. Consequently, no imputation of missing outcome data was performed. For time-to-event analysis, patients without documented failure were censored at the last available follow-up. The primary analyses were descriptive and longitudinal; the multivariable survival model was considered exploratory and was not intended to establish causal effects or comparative graft effectiveness.

To further characterize the prespecified age interval, outcomes were additionally summarized descriptively according to age group (40–49 and 50–60 years). These age-stratified analyses were considered descriptive and exploratory; no causal or comparative inference was intended.

## 3 Results

### 3.1 Study cohort

A total of 327 patients aged between 40 and 60 years underwent primary ACL reconstruction using non-irradiated frozen allograft tissue during the study period. After applying exclusion criteria and accounting for loss to follow-up, 300 patients constituted the final study cohort and were available for analysis. The mean age at the time of surgery was 48.6 ± 5.2 years (range, 40–60 years). The cohort included 182 men (60.7%) and 118 women (39.3%). The operated knee was the right side in 168 patients (56.0%) and the left side in 132 patients (44.0%). By design, all included patients had a pre-injury Tegner activity score between 4 and 7. The mean pre-injury Tegner score was 5.6 ± 1.2, reflecting a recreationally active cohort with moderate sporting demands rather than elite competitive participation.

The baseline demographic and clinical characteristics of the study cohort are summarized in Table 1.

**Table 1:** Baseline demographic and clinical characteristics of the study cohort (N = 300).

Variable	Value
Age at surgery, years (mean ± SD)	48.6 ± 5.2
Age range, years	40–60
Sex, n (%)	
• Male	182 (60.7)
• Female	118 (39.3)
Side of surgery, n (%)	
• Right	168 (56.0)
• Left	132 (44.0)
Follow-up duration, months (mean ± SD)	62.4 ± 9.1
Follow-up range, months	48–84
Concomitant meniscal procedure, n (%)	176 (58.7)
• Meniscal repair	94 (31.3)
• Partial meniscectomy	82 (27.3)
Advanced tibiofemoral osteoarthritis (KL ≥ 3)	0

The mean clinical follow-up was 62.4 ± 9.1 months (range, 48–84 months). Concomitant meniscal procedures were performed in 176 patients (58.7%), including meniscal repair in 94 patients (31.3%) and partial meniscectomy in 82 patients (27.3%). No patients had advanced tibiofemoral osteoarthritis at the time of surgery.

Regarding graft characteristics, tibialis anterior allografts were used in 168 patients (56.0%), tibialis posterior allografts in 84 patients (28.0%), and Achilles tendon allografts in 48 patients (16.0%). The mean prepared graft diameter was 9.1 ± 0.7 mm (range, 8.0–11.0 mm). Graft type and diameter distributions are summarized in Table 1.

### 3.2 Functional outcomes

Patient-reported outcome measures improved from preoperative assessment to final follow-up, with statistically significant within-patient differences observed for the IKDC subjective score and all KOOS domains (all p < 0.001).

The mean IKDC subjective score improved from 46.2 ± 11.8 preoperatively to 82.4 ± 10.6 at final follow-up. The mean KOOS overall score increased from 58.7 ± 13.4 preoperatively to 86.1 ± 9.8 postoperatively. The within-patient change in IKDC subjective score and the changes across all KOOS domains were statistically significant (all p < 0.001). The median postoperative Tegner activity score remained lower than the pre-injury activity level (p < 0.001).

Improvements were consistently observed across all KOOS subscales, including pain, symptoms, activities of daily living, sport and recreation, and quality of life. Preoperative and final follow-up patient-reported outcome measures are presented in Table 2.

**Table 2:** Patient-reported functional outcomes preoperatively and at final follow-up.

Outcome Measure	Baseline	Final Follow-up	p Value
IKDC subjective score	46.2 ± 11.8	82.4 ± 10.6	<0.001
KOOS Overall	58.7 ± 13.4	86.1 ± 9.8	<0.001
KOOS Pain	61.4 ± 14.1	89.3 ± 8.7	<0.001
KOOS Symptoms	59.2 ± 15.0	85.6 ± 10.1	<0.001
KOOS ADL	63.8 ± 12.9	90.4 ± 7.9	<0.001
KOOS Sport/Rec	42.5 ± 16.7	78.9 ± 12.4	<0.001
KOOS QOL	38.9 ± 15.6	76.8 ± 13.1	<0.001

Values are presented as mean ± standard deviation unless otherwise indicated. Within-patient changes in IKDC and KOOS scores were evaluated using paired-samples tests selected according to the distribution of paired differences. Tegner activity scores were compared using the Wilcoxon signed-rank test. ADL, activities of daily living; IKDC, International Knee Documentation Committee; KOOS, Knee injury and Osteoarthritis Outcome Score; MCID, minimal clinically important difference; QOL, quality of life; Sport/Rec, sport and recreation.

The mean Tegner activity score decreased from a pre-injury level of 5.6 ± 1.2 to 5.1 ± 1.1 at final follow-up. Despite this reduction, 219 patients (73.0% of the overall cohort) returned to their pre-injury recreational activity level or higher. Using the prespecified MCID threshold of 11.5 points for the IKDC subjective score, 271 patients (90.3%) demonstrated an individual improvement equal to or greater than this threshold from preoperative assessment to final follow-up.

### 3.3 Return to recreational sport participation

Return to recreational sport was achieved by 262 patients (87.3% of the overall cohort) during the follow-up period. The mean time to return to sport was 8.9 ± 2.4 months (range, 6–18 months). Among the 262 patients who returned to recreational sport, 219 (83.6%) resumed participation at their pre-injury activity level or higher, 29 (11.1%) returned at a reduced level, and 14 (5.3%) transitioned to lower-impact recreational activities different from their pre-injury sport. Thus, 219 patients (73.0% of the overall cohort) returned to their pre-injury recreational activity level or higher. Return-to-sport outcomes, including rates and time to return, are summarized in Table 3 and illustrated in Fig. 1.

**Table 3:** Resumption of recreational sport participation.

Variable	Value
Return to recreational sport, n (%)	262 (87.3)
Time to return to sport, months (mean ± SD)	8.9 ± 2.4
Time to return range, months	6–18
Return at pre-injury level or higher, n/N (%)	219/262 (83.6)
Return at reduced level, n/N (%)	29/262 (11.1)
Return to lower-impact activity, n/N (%)	14/262 (5.3)
No return to recreational sport, n (%)	38 (12.7)

Return to recreational sport participation was defined as documented resumption of recreational sporting activity for at least three consecutive months without interruption attributable to symptomatic instability or functional limitation of the operated knee. This outcome reflects sustained sport participation and should not be interpreted as formal return-to-sport readiness or clearance based on objective performance or psychological criteria. Percentages describing activity level after return were calculated among the 262 patients who resumed recreational sport participation.

**Fig. 1:**
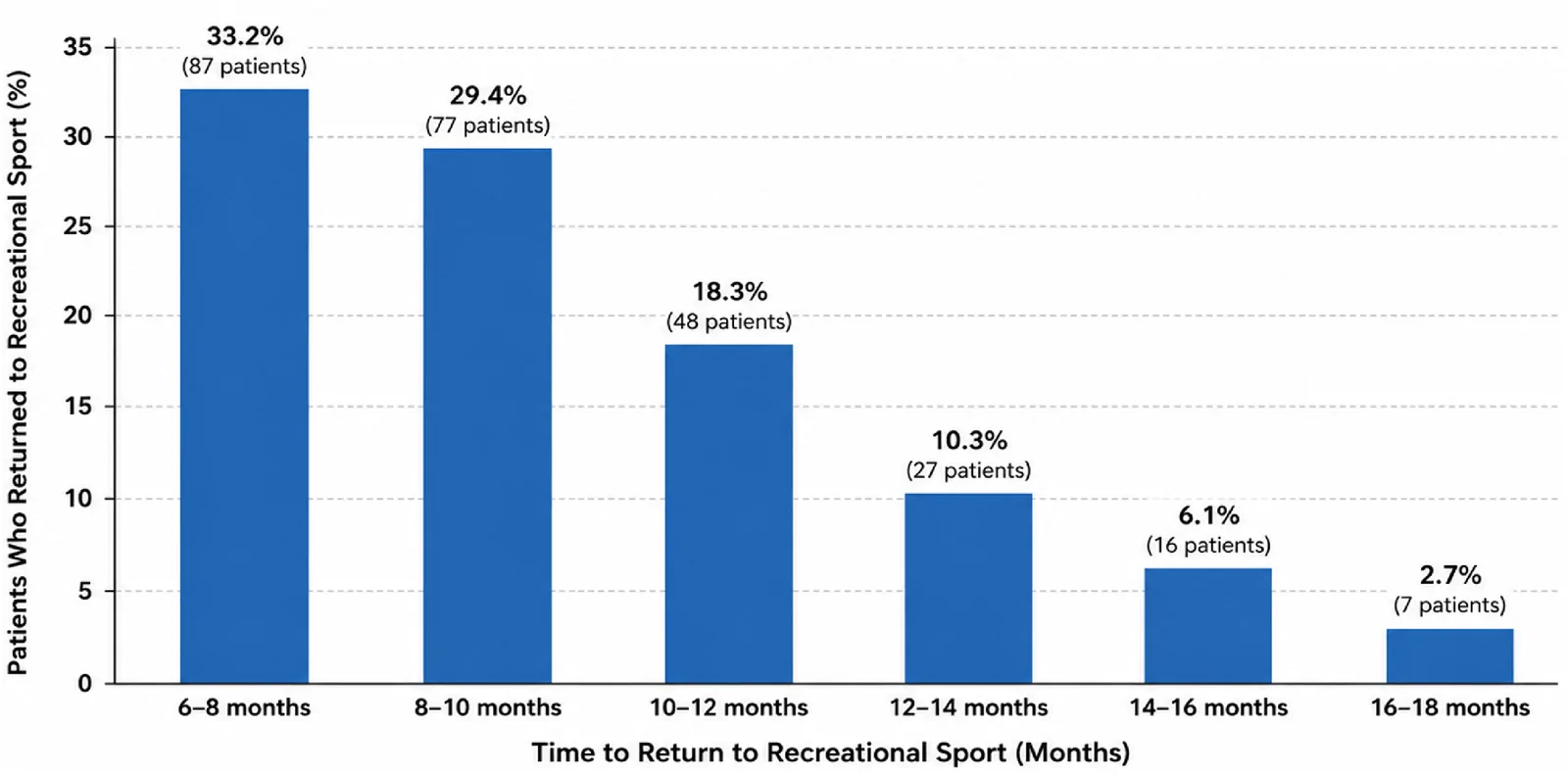
T**imeline of Functional Recovery and Return to Recreational Sport** Timeline depicting the distribution of time to return to recreational sport following anterior cruciate ligament reconstruction with non-irradiated frozen allograft. Time intervals are presented as mutually exclusive categories: 6–8 months, 8–10 months, 10–12 months, 12–14 months, 14–16 months, and 16–18 months. Percentages were calculated among the 262 patients who returned to recreational sport.

Thirty-eight patients (12.7%) did not return to recreational sports. Reasons documented in clinical records included persistent knee symptoms without objective instability, lifestyle changes unrelated to knee function, or personal preference.

### 3.4 Graft survivorship

Kaplan–Meier survival analysis demonstrated favorable graft survivorship over the follow-up period. Estimated graft survivorship was 98.3% at 2 years, 96.7% at 3 years, and 94.8% at 5 years. Corresponding 95% confidence intervals were derived from the Kaplan–Meier estimator and are reported in Table 4 and Fig. 2. A total of 16 graft failures (5.3%) were recorded during the study period. The mean time to graft failure was 32.6 ± 14.1 months following surgery. Graft survivorship over the follow-up period is shown using Kaplan–Meier analysis, with the curve starting at 100% survivorship at time zero and estimated survivorship of 98.3%, 96.7%, and 94.8% at 2, 3, and 5 years, respectively (Fig. 2).

**Table 4:** Graft survivorship and postoperative complications.

Outcome	Value
Graft failures, n (%)	16 (5.3)
Mean time to graft failure, months (mean ± SD)	32.6 ± 14.1
Kaplan–Meier graft survivorship	
• 2 years	98.3%
• 3 years	96.7%
• 5 years	94.8%
Reoperations (excluding revision ACL), n (%)	21 (7.0)
• Cyclops lesion	9 (3.0)
• Hardware removal	7 (2.3)
• Meniscal reintervention	5 (1.7)
Postoperative stiffness requiring MUA, n (%)	4 (1.3)
Deep infection	0
Thromboembolic events	0
Allograft-related adverse reactions	0

**Fig. 2:**
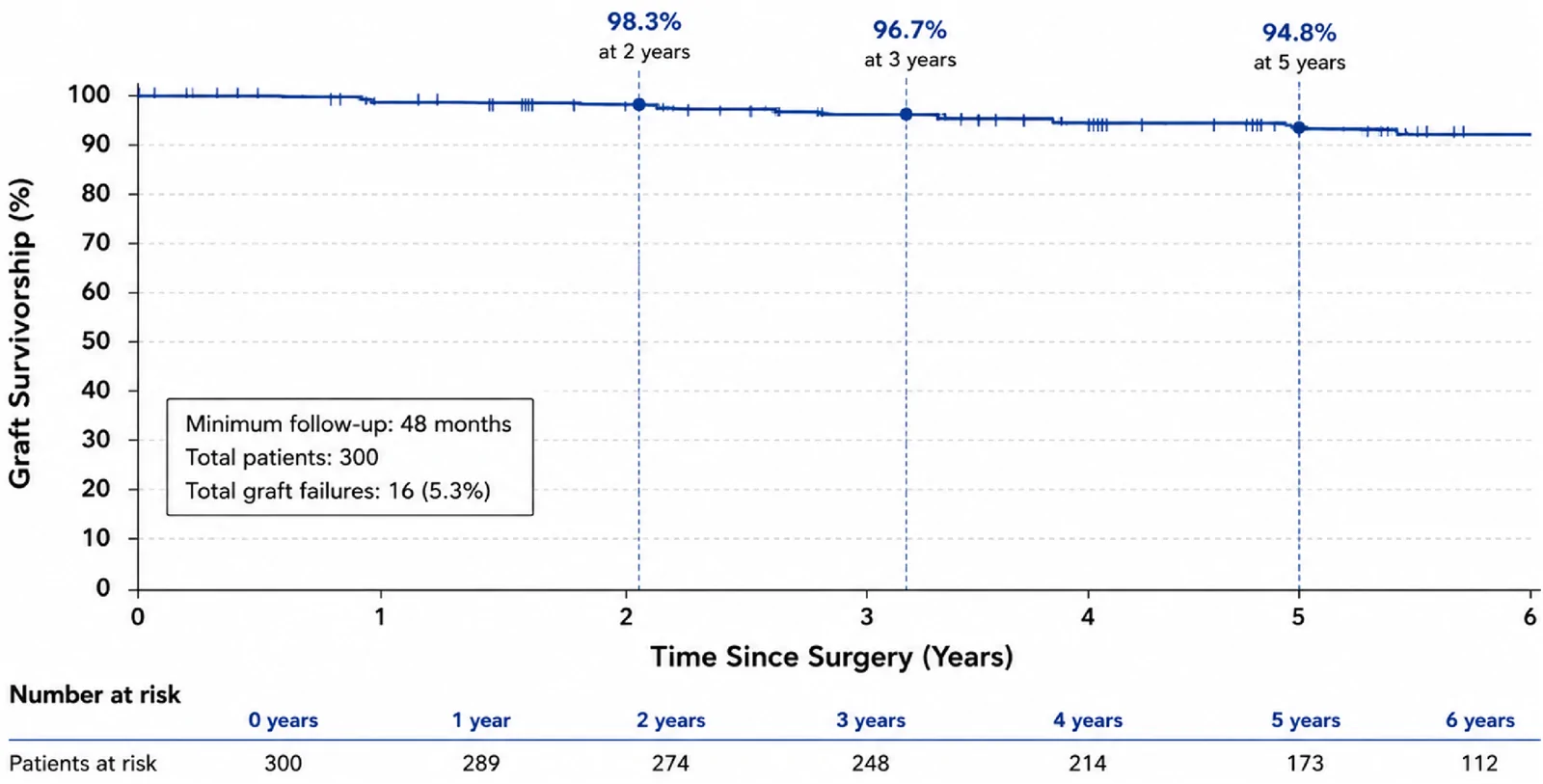
**Kaplan–Meier Analysis of Graft Survivorship** Kaplan–Meier survival curve illustrating graft survivorship after primary anterior cruciate ligament reconstruction using non-irradiated frozen allograft in recreational athletes aged 40–60 years. The curve begins at 100% survivorship at time zero. Estimated graft survivorship was 98.3% at 2 years, 96.7% at 3 years, and 94.8% at 5 years. Tick marks indicate censored observations.

Patients who experienced graft failure were censored at the time of revision surgery for survivorship analysis. No clustering of failures was observed during the early postoperative period.

### 3.5 Exploratory multivariable survival analysis

Given the limited number of graft-failure events, adjusted associations with graft failure were examined using penalized Cox proportional hazards regression. Age at surgery, sex, pre-injury Tegner activity score, concomitant meniscal procedure, and prepared graft diameter were included as prespecified covariates. The adjusted model was considered exploratory and was used to assess the stability of the observed survivorship findings after accounting for clinically relevant baseline and surgical characteristics. No violation of the proportional hazards assumption was identified on assessment of Schoenfeld residuals.

### 3.6 Complications

Graft rerupture occurred in 16 patients (5.3%), all of whom underwent revision ACL reconstruction. Reoperations unrelated to graft failure were performed in 21 patients (7.0%), including arthroscopic treatment for cyclops lesions (n = 9), symptomatic hardware removal (n = 7), and meniscal reintervention (n = 5). Postoperative stiffness requiring manipulation under anesthesia occurred in 4 patients (1.3%). Postoperative complications and reoperations are detailed in Table 4.

No cases of deep infection, thromboembolic events, or graft-related immunological reactions were observed. There were no reported cases of disease transmission associated with allograft use.

## 4 Discussion

### 4.1 Principal finding

The principal finding of this study was that primary allograft anterior cruciate ligament reconstruction in recreationally active adults aged 40–60 years was associated with substantial functional improvement, high rates of return to recreational sport, and satisfactory graft survivorship at mid-term follow-up. Across a large, consecutively treated cohort, patient-reported outcome measures improved substantially from preoperative assessment to final follow-up, a high proportion of patients achieved the prespecified published MCID threshold for the IKDC subjective score, documented resumption of recreational sport participation occurred in the majority of patients, and graft failure rates remained within an acceptable range over a minimum five-year period. These findings provide mid-term outcome data in a well-defined patient population for whom evidence has historically been limited. 7, 8, 9, 10.

### 4.2 Clinical interpretation

From a clinical perspective, the results provide mid-term outcome estimates for allograft ACL reconstruction in selected recreationally active adults aged 40–60 years. Because graft processing was uniform across the cohort and no alternative processing or graft-source group was evaluated, the findings should not be interpreted as evidence of superiority of non-irradiated frozen grafts over other contemporary allograft preparations or autografts.

The return-to-sport findings require specific interpretation. The outcome used in this study captured sustained resumption of recreational sport participation and was not designed to establish objective return-to-sport readiness. Contemporary return-to-sport assessment after anterior cruciate ligament reconstruction increasingly incorporates multidimensional criteria, including strength recovery, functional performance, movement quality, and psychological readiness. Because these measures were not systematically available in the retrospective records, the 87.3% participation rate should not be interpreted as the proportion of patients who satisfied comprehensive return-to-sport clearance criteria. Rather, it represents the proportion with documented sustained resumption of recreational sporting activity according to the operational definition used in this study.

For the treating surgeon, the observed combination of functional recovery, return to activity, and graft durability supports its use in patients whose primary objectives are restoration of knee stability and sustained participation in recreational sports rather than return to high-level competitive performance. The high proportion of patients achieving the prespecified IKDC MCID threshold, together with the observed return-to-sport outcomes, indicates favorable functional recovery within this selected cohort. 11, 12, 13, 14, 15, 16.

At the same time, these findings should be interpreted within the context of appropriate patient selection. The population studied consisted of individuals with clearly defined recreational activity demands, preserved joint status, and no advanced degenerative changes at the time of surgery. While the results support the use of non-irradiated frozen allograft in such patients, they may not necessarily be generalizable to younger athletes engaged in pivoting sports at high competitive levels, or to patients with complex multiligamentous injuries or substantial chondral compromise. In these scenarios, graft choice must continue to be individualized based on patient characteristics, activity demands, and surgeon judgment. 15, 16, 17, 18, 19, 20.

### 4.3 Comparison with existing literature

The present findings are consistent with prior reports describing acceptable outcomes of ACL reconstruction in middle-aged populations, where functional improvement and patient satisfaction have been shown to be achievable despite increasing age. Previous studies evaluating allograft use in ACL reconstruction have reported variable results, often influenced by differences in graft processing, patient age, and activity level. In particular, concerns regarding higher failure rates have frequently been associated with irradiated or chemically processed grafts and with younger, high-demand cohorts. 21, 22, 23, 24, 25.

The present cohort was characterized by uniform use of non-irradiated frozen allograft tissue. This processing profile should primarily be considered a methodological characteristic of the study rather than its principal source of novelty, particularly because similar processing strategies are routinely used in many contemporary settings. The clinical contribution of the present study lies instead in the characterization of mid-term functional recovery, return to recreational sport, and graft survivorship in a defined age and activity population that remains less frequently isolated in the anterior cruciate ligament reconstruction literature. Importantly, the study was not designed to establish superiority of one graft source or processing strategy over another. The graft survivorship observed in the current cohort aligns with published mid-term data in comparable age groups, while functional scores and return-to-sport rates are similar to those reported in series emphasizing patient-reported outcomes rather than competitive performance metrics. Importantly, the present study does not seek to establish superiority of one graft option over another, but rather to contextualize the outcomes associated with a specific graft choice in a specific clinical scenario. 26, 27, 28, 29, 30.

### 4.4 Strengths

Several strengths of this study merit consideration. First, the cohort size is substantial for a single-center series focusing on a narrowly defined age and activity subgroup, allowing for more precise estimation of mid-term outcomes and graft survivorship. Second, the minimum follow-up of five years provides insight beyond short-term recovery and captures clinically relevant events such as graft failure and reoperation. Third, recreational athlete status was operationalized using both the absence of elite competitive participation and a prespecified pre-injury Tegner activity range of 4 to 7, providing a more objective characterization of baseline activity demand. Finally, the use of validated patient-reported outcome measures and a clearly defined graft failure endpoint strengthens the reliability of the reported results. 31, 32, 33, 34, 35.

### 4.5 Limitations

This study has several limitations that should be acknowledged. Its retrospective design introduces inherent limitations related to data collection and potential unmeasured confounding factors. Although an exploratory penalized multivariable survival analysis was performed to account for selected baseline and surgical characteristics, only 16 graft-failure events occurred. The limited number of events constrained model complexity and statistical precision, and residual confounding by unmeasured factors, including sport-specific demand and detailed rehabilitation exposure, remains possible. Accordingly, the adjusted hazard estimates should be interpreted as exploratory rather than confirmatory.

The absence of a contemporaneous control group precludes direct comparison with alternative graft options and limits causal inference regarding graft selection. Selection bias is also possible, as graft choice may have been influenced by patient characteristics, surgeon preference, or shared decision-making, which cannot be fully accounted for in this analysis. 36, 37, 38, 39, 40.

The age boundaries used in this study were operational and should not be interpreted as a universal definition of middle age. Although the 40–60-year interval was prespecified to characterize an older recreationally active anterior cruciate ligament reconstruction population, chronological age alone does not fully capture biological age, sport-specific demand, or degenerative joint burden. The age-stratified analyses were descriptive, and the study was not powered to identify age-by-treatment interactions or determine an optimal age threshold for allograft selection.

Additionally, while the cohort was well defined, the findings may not be generalizable to populations with different activity levels, younger patients, or those with advanced degenerative joint changes. Although the pre-injury Tegner activity range provided objective activity-based stratification, residual heterogeneity in sport type and biomechanical demand remained within the recreational athlete population. In particular, the present analysis did not stratify outcomes according to participation in pivoting versus non-pivoting or low-impact sports. Accordingly, differences in sport-specific loading may have influenced return-to-sport and graft survivorship estimates and should be addressed in future subgroup analyses. The assessment of return to recreational sport represents an important limitation. Return was determined from clinical documentation and patient report and was operationally defined according to sustained sport participation rather than a standardized multidimensional return-to-sport battery. Objective strength testing, hop-test performance, limb-symmetry criteria, movement-quality assessment, and validated psychological readiness measures were not systematically available across the study period. Consequently, the reported 87.3% rate may overestimate the proportion of patients who would have fulfilled contemporary criteria for formal return-to-sport readiness or clearance. In addition, psychological barriers, including fear of reinjury and reduced confidence, could not be quantified using a validated instrument such as the Anterior Cruciate Ligament–Return to Sport after Injury scale. The return-to-sport findings should therefore be interpreted as rates of documented sustained recreational sport participation rather than evidence of comprehensive functional or psychological readiness. Finally, although mid-term follow-up was achieved for all included patients, longer-term outcomes beyond five years were not evaluated and may reveal additional insights regarding graft durability and joint health. 41, 42, 43, 44, 45, 46.

In addition, graft processing was uniform across the cohort, and no irradiated, chemically processed, or alternative preservation group was available for comparison. The study therefore cannot determine whether the observed outcomes were attributable to the non-irradiated frozen processing profile or whether similar results would have been achieved with other contemporary allograft preparations.

## 5 Conclusion

In recreationally active adults aged 40–60 years, primary allograft anterior cruciate ligament reconstruction was associated with substantial improvement in patient-reported outcomes, high rates of return to recreational sport, and satisfactory graft survivorship at mid-term follow-up. These findings provide outcome estimates for a defined older recreationally active population treated with a consistent allograft-processing protocol. Because no alternative graft source or processing method was evaluated, the results should not be interpreted as evidence of comparative superiority of non-irradiated frozen allograft.

Within this clearly defined patient population, substantial improvements in patient-reported outcomes were observed, and most patients achieved the prespecified published MCID threshold for the IKDC subjective score, while graft failure and reoperation rates remained acceptable over a minimum five-year period.

## Ethical approval

The study was approved by the Institutional Review Board of Clinica Arthros, under approval number ARTC-00146. All study procedures were conducted in accordance with the applicable institutional ethical standards and the principles of the Declaration of Helsinki.

## Author contributions (CRediT taxonomy)

Conceptualization: Horacio Rivarola, Francisco Endara Urresta.

Methodology: Horacio Rivarola, Cristian Collazo, Francisco Endara Urresta.

Formal Analysis: Horacio Rivarola, Marcos Palanconi.

Investigation: Cristian Collazo, Marcos Meninato, Carlos Peñaherrera-Carrillo, Alejandro Barros Castro, Brian Flores, Bautista Rivarola.

Data Curation: Marcos Meninato, Alejandro Barros Castro.

Writing – Original Draft: Horacio Rivarola, Francisco Endara Urresta.

Writing – Review & Editing: Cristian Collazo, Marcos Palanconi, Carlos Peñaherrera-Carrillo.

Visualization: Marcos Palanconi, Francisco Endara Urresta.

Supervision: Horacio Rivarola, Francisco Endara Urresta.

Project Administration: Horacio Rivarola.

## Funding

No external funding was received for this study.

## Declaration of competing interest

The authors declare that they have no known competing financial interests or personal relationships that could have appeared to influence the work reported in this paper.
