# Supplementary material for: Mid-term functional outcomes and graft survivorship after allograft ACL reconstruction in recreationally active adults aged 40–60 years
Source: Asia Pac J Sports Med Arthrosc Rehabil Technol. 2026 Nov 7;46:109–16. doi: 10.1016/j.asmart.2026.10.002 (PMC13769469; doi:10.1016/j.asmart.2026.10.002)
Supplement: Supplementary file 1 [file mmc1.docx]

# STROBE Checklist – Completed

Title and Abstract
1a. Study design indicated in title/abstract – Yes
1b. Informative and balanced abstract – Yes

Introduction
2. Background and rationale explained – Yes
3. Objectives clearly stated – Yes

Methods
4. Study design presented early – Yes
5. Setting, locations, and dates described – Yes
6a. Eligibility criteria and participant selection described – Yes
6b. Matching criteria – Not applicable
7. Variables clearly defined – Yes
8. Data sources and measurements described – Yes
9. Bias addressed – Yes
10. Study size explained – Yes
11. Quantitative variables handled appropriately – Yes
12a. Statistical methods described – Yes
12b. Subgroup analyses – Not applicable
12c. Missing data addressed – Yes
12d. Loss to follow-up addressed – Yes
12e. Sensitivity analyses – Not applicable

Results
13a. Participant numbers at each stage reported – Yes
13b. Reasons for non-participation – Yes
13c. Flow diagram – Considered
14a. Descriptive data provided – Yes
14b. Missing data reported – Yes
14c. Follow-up time summarized – Yes
15. Outcome data reported – Yes
16a. Main results reported – Yes
16b. Category boundaries reported – Yes
16c. Absolute risk translation – Not applicable
17. Other analyses – Not applicable

Discussion
18. Key results summarized – Yes
19. Limitations discussed – Yes
20. Interpretation cautious and balanced – Yes
21. Generalisability discussed – Yes

Other Information
22. Funding source declared – Yes
